# Ethical challenges in treating family members: A case study on confidentiality and clinical decision-making in the Indian context

**DOI:** 10.1177/2050313X251380521

**Published:** 2025-09-28

**Authors:** Mohamed Ali Kalathingal, Nazia Edathola Kottasseri, Jasim Uluvan, Shajitha Thekke Veettil

**Affiliations:** 1 Family Medicine Clerkship Office, Primary Health Care Corporation, Doha, Qatar; 2 Qatar University Health Centre, Primary Health Care Corporation, Doha, Qatar; 3 Department of Medicine, Relief Hospital, Kondotty, Kerala, India; 4 Department of Clinical Research, Primary Health Care Corporation, Doha, Qatar

**Keywords:** physician ethics, confidentiality, family care, IgA nephropathy, dual-role conflict, Indian healthcare

## Abstract

This case report explores the ethical complexities faced by physicians when providing medical advice and care to close family members. It focuses on a 29-year-old Indian woman residing in Western Europe who, while visiting family in Kerala, India, was diagnosed with IgA nephropathy during a routine health check. Several family members involved in her care are also practicing physicians, raising significant ethical questions about confidentiality, professional objectivity, and the boundaries of familial care. The case highlights the tension between patient autonomy and informal medical involvement by relatives, especially in a country such as India, where cultural expectations encourage familial responsibility. Although clinical care was efficiently delivered, the situation raised concerns about the formal decision-making process, the lack of clinical documentation, and the absence of established ethical frameworks within the Indian healthcare context. This report emphasizes the need for clearer guidelines and practical tools to help physicians navigate dual-role relationships while upholding confidentiality and clinical integrity. The patient’s perspective and the follow-up are discussed, with implications for both policy and practice.

## Introduction

The ethical responsibility to uphold patient confidentiality is a cornerstone of clinical care. This duty becomes significantly more complex when physicians are asked to treat or advise close family members. In such cases, the boundaries between personal and professional roles blur, increasing the risk of compromised objectivity, unintentional breaches of confidentiality, and emotionally driven decision-making.^1^

Internationally, several regulatory bodies discourage or prohibit physicians from treating family members due to these risks. The General Medical Council (GMC UK),^2^ American Medical Association (AMA),^3–5^ and the College of Physicians and Surgeons of Ontario (CPSO)^6^ emphasize the need to maintain professional distance to ensure unbiased medical care. Appropriate “personal and professional boundaries” are essential between medical professionals and their patients, including relatives, and between medical professionals and their colleagues^2,3^ to avoid conflicts of interest. According to CPSO, physicians are strongly advised not to treat themselves or close family members, except “in limited circumstances, such as for minor conditions, in emergencies, or when another qualified health professional is not readily available.” The policy emphasizes that “objectivity, patient autonomy, and professional judgment may be compromised” when treating those with whom the physician has a close personal relationship.^7^ However, India lacks such formal restrictions, allowing for greater ambiguity in physician-family interactions.^8,9^

In the Indian sociocultural context, familial involvement in healthcare is not only common but often expected. Trust in relatives who are medical professionals is high, and informal consultations are frequently sought during family gatherings or emergencies.^10^ While these practices are culturally deep-rooted, they create ethical gray zones in terms of professional conduct and patient confidentiality.^11^ Although the 2002 Code of Medical Ethics by the National Medical Commission (NMC) imposes duties of confidentiality and record-keeping even for family consultations, in a regulatory context.^12^ As per this policy, every physician shall maintain the medical records pertaining to his/her indoor patients for a period of 3 years from the date of commencement of the treatment in a standard proforma laid down by the Medical Council of India.^12^

This report presents a case that exemplifies these ethical tensions. A young Indian woman, visiting from Europe, underwent diagnostic testing during a family visit to Kerala and was found to have significant renal pathology. Several of her relatives, who are physicians, became involved in her diagnostic and treatment pathway. This case study aims to examine how the ethical principles of confidentiality and professional boundaries were managed in this context, with a focus on practical challenges, cultural influences, and the absence of formal ethical guidelines in India.

## Case presentation

The patient was a 29-year-old Indian woman, previously healthy and residing in Western Europe. She had no known past medical history, was not on any regular medications, and had no significant family history of kidney disease or other chronic illnesses. During a family visit to Kerala, India, she decided to undergo a routine health check-up—not due to any specific symptoms, but simply to assess her overall health. This decision was influenced by the availability of high-quality, affordable health screening services in India, and the opportunity to seek informal advice from relatives, many of whom are medical professionals.

Unexpectedly, her initial laboratory results revealed several abnormalities:

- *Hemoglobin*: 10.6 g/dL (normal: 12–15.5 g/dL)—indicating mild anemia.
- *Blood urea:* 51.9 mg/dL (normal: 7–20 mg/dL)—mildly elevated, possibly from dehydration.
- *Serum creatinine:* 1.82 mg/dL (normal: 0.6–1.1 mg/dL for females)—suggestive of renal impairment.
- *Urine analysis:* 3+ proteinuria—indicative of possible glomerular disease.
- *Lipid profile:* Elevated total cholesterol and LDL, likely diet-related.

Despite being completely asymptomatic, these unexpected findings prompted concern. The initial results were shared informally via WhatsApp with a relative physician practicing abroad. He recommended repeating the tests and pursuing further renal investigations.

Follow-up assessments, including ultrasound of the kidneys, ureters, and bladder, and a urine microalbumin/creatinine ratio, confirmed persistent abnormalities. The patient was referred to a tertiary hospital where a nephrologist recommended a renal biopsy. Within 5 days, a biopsy confirmed IgA nephropathy, a chronic glomerular disease. The diagnosis was communicated sensitively, and treatment was discussed in consultation with specialists and family physicians. Though the patient had access to care locally and family support in India, she elected to return to Europe to initiate long-term nephrology care. Her decision was guided by a desire for continuity under her insurance coverage and personal confidentiality.

Throughout this process, several family members were involved in interpreting lab results, suggesting specialists, and discussing treatment options. While well-intentioned, this dynamic introduced a dual-role dilemma: relatives were simultaneously acting as caregivers, family supporters, and informal medical advisors. This raised concerns about professional boundaries, confidentiality, and objectivity in clinical decision-making.

## Discussion

The core ethical challenge in this case revolves around the blurred boundaries between personal and professional roles when physicians are involved in the care of close family members. Although the involvement of family members was well-intentioned and medically helpful, it raises key ethical concerns—particularly regarding confidentiality, objectivity, and informed decision-making.

International ethical frameworks, such as those from the GMC,^2^ AMA,^3–5^ and others, discourage physicians from treating or advising close relatives except in emergencies. The rationale is to prevent emotional involvement from compromising clinical judgment, ensure patient privacy, and avoid situations where patients may withhold sensitive information.^2,3,13^ Beigel et al., in their systematic review, noted that physicians frequently provide care to family members and friends, despite ethical guidelines advising against it. The review highlights both the motivations for and concerns about treating loved ones, while also critiquing existing ethical frameworks for offering insufficient practical guidance. The ethical complexities surrounding this practice warrant more thorough consideration.^14^

In this case, the patient’s initial diagnostic process was initiated and guided through informal channels, including advice over messaging apps and test interpretations by relatives. While effective in coordinating rapid care, it bypassed formal documentation, potentially compromised confidentiality, and placed undue pressure on both the patient and her physician relatives.^15–18^ Such practices can lead to difficulties in ensuring unbiased care, especially if the physician-families rely on assumptions or shared history rather than standard protocols.

Notably, the patient was asymptomatic, yet significant findings prompted urgent medical decisions. While care was appropriate and timely, the lack of structured clinical guidelines or oversight—common in informal consultations—may have exposed the patient to unnecessary anxiety or unclear consent procedures. This risk is particularly heightened in contexts like India, where there are no explicit professional restrictions against treating family members,^8^ and cultural expectations often support familial responsibility in healthcare decisions.^10^

A key issue in this case is the clinical rationale for performing a renal biopsy in an asymptomatic patient. Although the patient reported no overt symptoms, laboratory findings—including elevated serum creatinine (1.82 mg/dL) and 3+ proteinuria—were strongly suggestive of underlying glomerular disease. According to international nephrology guidelines, particularly those from Kidney Disease: Improving Global Outcomes, a biopsy is indicated in patients with unexplained proteinuria and impaired renal function, even in the absence of clinical symptoms. This approach enables accurate diagnosis, such as identifying IgA nephropathy, and allows for timely therapeutic decisions aimed at preserving long-term renal function. In this case, the nephrologist’s decision was guided by these established criteria to prevent diagnostic delay and to tailor appropriate treatment.^19^

The decision-making process was collaborative, involving multiple family members and external nephrologists. While this demonstrated dedication, it also risked groupthink and relational pressure, as relatives who had experienced kidney disease offered unsolicited advice or promoted particular specialists based on their past experiences—some of which were clinically irrelevant to this patient’s condition.

The issue of patient confidentiality was central yet subtle. Although the patient consented to her relatives’ involvement, it remains unclear whether she fully understood the implications of sharing medical details within a family network. Furthermore, the informal sharing of medical data through WhatsApp—while practical—raises concerns about privacy, particularly in cross-border contexts involving multiple healthcare systems, though such practices are not compliant with HIPAA/GDPR standards.^20,21^ WhatsApp, though commonly used by clinicians, is not compliant with HIPAA or GDPR due to the absence of a Business Associate Agreement, audit trails, access controls, and medical record integration. UK ICO and NHS England state that any patient data shared via WhatsApp must be securely documented and deleted from personal devices. GDPR further prohibits handling sensitive health data without proper safeguards. While permitted in emergencies or if initiated by patients, explicit consent, privacy warnings, and secure documentation are essential. In this case, WhatsApp use raised concerns about confidentiality, data security, and legal noncompliance with international standards.^20,21^ Physicians must exercise caution and avoid using platforms such as WhatsApp for clinical communications unless appropriate consent, documentation, and data governance protocols are in place.^20–22^

Despite these concerns, the case also highlights Kerala’s efficient healthcare access, affordability, and diagnostic infrastructure. These factors enabled the rapid detection and confirmation of IgA nephropathy, which might have taken longer in more resource-constrained systems abroad.^23^

## Cultural factors

In many South Asian settings, including India, familial involvement in healthcare is deeply rooted in cultural expectations. Trust in physician family members is often seen as a natural extension of care and support, especially during medical uncertainty. In this case, the patient’s choice to undergo a health check-up while visiting her family in Kerala reflects a culturally endorsed reliance on kinship for both emotional and clinical guidance. While this support can be beneficial, it also blurs professional boundaries, making it difficult for physicians to maintain objectivity or ensure that consent processes are free of implicit pressure or familial influence.^7,14^ Moreover, informal communication platforms such as WhatsApp are widely used for rapid coordination, often viewed as pragmatic in resource-rich, culturally tight-knit environments, but not necessarily ethical or secure.

## Legal framework

From a legal and regulatory perspective, the Indian healthcare system currently lacks specific, enforceable restrictions on physicians treating close family members. However, the 2002 Code of Medical Ethics issued by the NMC mandates duties of confidentiality and proper record-keeping that are applicable even in informal settings. International guidelines, such as those from the GMC and the CPSO, explicitly discourage such dual-role relationships except in emergencies.^7^ Furthermore, the informal use of WhatsApp to share patient results raises serious concerns regarding compliance with HIPAA and GDPR, both of which require secure data handling, audit trails, and informed consent.^19–21^ These gaps underscore the need for Indian regulatory bodies to establish clear ethical standards and digital communication protocols to safeguard patient autonomy and clinical accountability, especially in culturally sensitive scenarios.

## Limitations

One limitation of this case report is the absence of direct documentation from the patient’s perspective regarding her experience of being treated or advised by family physicians. Future studies or case analyses should include this critical viewpoint to better understand how patients perceive the trade-offs between familial involvement and confidentiality.

## Practical recommendations

1. *Avoid informal consultation* for family members beyond initial triage or emergencies. Encourage use of independent healthcare providers for full evaluation and management.
2. *Respect patient confidentiality*, even in familial contexts. Avoid sharing medical information through unsecured channels or without explicit, informed consent.
3. *Document clinical decisions* thoroughly, even when care is informally provided. This helps protect both the patient and the provider from future ethical or legal issues.
4. *Raise awareness among physicians*, particularly in countries without formal regulations—about the ethical risks of treating relatives and the importance of maintaining professional boundaries.

Further research in similar cultural contexts is needed to explore patients’ experiences.

## Patient’s perspective

The patient’s subjective experience and reflections on receiving informal medical advice from family members were not directly recorded in this case. This omission is acknowledged as a limitation, and future reports should aim to capture the patient voice to fully understand the ethical dimensions involved.

## Conclusion

This case highlights the complex ethical terrain that physicians must navigate when involved in the care of close family members. While the patient ultimately received timely and appropriate treatment for IgA nephropathy, the process raised concerns about professional objectivity, patient confidentiality, and informal medical decision-making.

The blurred lines between familial duty and clinical responsibility can lead to unintended ethical compromises—especially when care is initiated outside formal systems. In this case, the absence of clear professional boundaries and the use of informal communication channels risked undermining patient autonomy and privacy, even if no harm occurred.

Despite its challenges, this case underscores the importance of applying universal ethical standards—such as those outlined by the GMC and AMA—regardless of cultural or local regulatory gaps. Physicians must carefully weigh the risks of dual-role relationships and consider deferring care to an independent provider whenever possible. The ethical complexities surrounding this practice warrant more thorough consideration.
